# O.5.3-2 Development of a framework for scaling up community-based health promotion: a best fit framework synthesis

**DOI:** 10.1093/eurpub/ckad133.253

**Published:** 2023-09-11

**Authors:** Philipp Weber, Leonie Birkholz, Natalie Helsper, Lea Dippon, Simone Kohler, Klaus Pfeifer, Alfred Rütten, Jana Semrau

**Affiliations:** Friedrich-Alexander-Universität Erlangen-Nürnberg; leonie.birkholz@fau.de; Friedrich-Alexander-Universität Erlangen-Nürnberg; leonie.birkholz@fau.de; Friedrich-Alexander-Universität Erlangen-Nürnberg; leonie.birkholz@fau.de; Friedrich-Alexander-Universität Erlangen-Nürnberg; leonie.birkholz@fau.de; Friedrich-Alexander-Universität Erlangen-Nürnberg; leonie.birkholz@fau.de; Friedrich-Alexander-Universität Erlangen-Nürnberg; leonie.birkholz@fau.de; Friedrich-Alexander-Universität Erlangen-Nürnberg; leonie.birkholz@fau.de; Friedrich-Alexander-Universität Erlangen-Nürnberg; leonie.birkholz@fau.de

## Abstract

**Purpose:**

Community-based health promotion with a focus on people with social disadvantages is essential to address persistently existing health inequities. However, achieving an impact on public health requires scaling up such approaches beyond manifold funded pilot projects. The aim of this review is to provide an overview of scaling-up frameworks in health promotion and to identify key components for scaling up community-based health promotion.

**Methods:**

We followed the “best fit” framework synthesis approach and conducted a systematic search for scaling-up frameworks for health promotion in PubMed, CINAHL, Scopus, Web of Science, PsycInfo, and SportDiscus. Based on the included frameworks, we created an a priori framework. Second, we searched for primary research studies in the same databases that reported scaling-up processes of community-based health promotion. We coded the data using the a priori framework.

**Results:**

From 80 articles, 5 were included for data extraction. The analysis yielded 10 a priori defined key components: “innovation characteristics”; “clarify and coordinate roles and responsibilities”; “build up skills, knowledge, and capacity”; “mobilize and sustain resources”; “initiate and maintain regular communication”; “plan, conduct, and apply assessment, monitoring, and evaluation”; “develop political commitment and advocacy”; “build and foster collaboration”; “encourage participation and ownership”; and “plan and follow strategic approaches”. We further identified 113 primary research studies; 10 were eligible. No new key components were found, but all a priori defined key components were supported by the studies. Ten key components for scaling up community-based health promotion represent the final framework.

**Conclusion:**

The “best fit” framework synthesis approach provides science-based insights for developing successful scaling up processes in the field of physical activity-related health promotion. We further identified “encourage participation and ownership” as a crucial component regarding health equity. Moreover, practice-based evidence from different stakeholders at various levels will be needed to develop a practice and science-based concept to scaling up a community-based health promotion approach.

**Funding Source:**

This research was funded by the Federal Centre of Health Education (BZgA) on behalf of and with funds from the statutory health insurances according to § 20a SGB V in the context of the GKV Alliance for Health.

